# Linking disease registries and nationwide healthcare administrative databases: the French renal epidemiology and information network (REIN) insight

**DOI:** 10.1186/s12882-020-1692-4

**Published:** 2020-01-28

**Authors:** Maxime Raffray, Sahar Bayat, Mathilde Lassalle, Cécile Couchoud

**Affiliations:** 1 Univ Rennes, Ecole des Hautes Etudes en Santé Publique (EHESP), Recherche en pharmaco-épidémiologie et recours aux soins (REPERES) – EA 7449, 15 Avenue du Professeur Léon Bernard, 35043 Rennes, France; 2 Coordination nationale Registre REIN, Agence de la biomédecine, 1, avenue du Stade-de-France, 93212 Saint-Denis-La-Plaine cedex, France

## Abstract

**Background:**

Record linkage is increasingly used in health research worldwide. Combining the patient information available in healthcare, administrative and clinical databases broadens the research perspectives, particularly for chronic diseases. Recent guidelines highlight the need for transparency on the used record linkage processes and the extracted data to be used by researchers.

**Methods:**

Therefore, the aim of this study was to describe the deterministic iterative approach used to link the French Epidemiology and Information Network (REIN), a French national End-Stage Renal Disease registry, with the Système National des Données de Santé (SNDS), a French nationwide medico-administrative healthcare database.

**Results:**

Among the 22,073 patients included in the REIN registry who started renal replacement therapy between 2014 and 2015 in France, 19,223 (87.1%) were matched with patients in the SNDS database. Comparison of matched and unmatched patients confirmed the absence of any major selection bias. Then, the record linkage was evaluated using the comorbidity status (diabetes).

**Conclusions:**

This fast and efficient method of record linkage with pseudonymized data and without unique and direct identifier might inspire other research teams. It also opens the path for new research on chronic kidney disease.

## Background

Record linkage is an increasingly important tool for public health research and epidemiology [1–3]. Indeed, linking databases increases the information available on each patient (clinical and administrative data, disease-related mortality, healthcare utilization…) and consequently broadens the research opportunities [4–9]. Record linkage is especially relevant for chronic and multifactorial diseases, such as Chronic Kidney Disease (CKD), because it allows a more comprehensive understanding of the risk factors and outcomes [10–14]. For example, inequalities related to access to renal transplantation were recently investigated by combining individual-level socioeconomic and clinical data [15].

Worldwide, many population-based databases have been built by linking different databases, with diverse methods [16–19]. Consequently, recent publications underlined the necessity for a greater transparency about the production and the use of linked data in health research [1, 20]. The 2017 GUidance for Information about Linking Datasets (GUILD) recommends sharing information about the linkage process. The aim is to allow researchers using these data to be aware of potential biases, thus improving the interpretation of results based on linked data and their overall quality [1].

The French healthcare system has one central administrative database (Système National des Données de Santé [SNDS]; National System of Health Data) that includes all ambulatory care and hospital stay reimbursement data nation-wide and also death-related data [21]. However, the reimbursement data it collects were not originally for research purposes, and for example, precise clinical data, such as the stage or severity of the disease or comorbid conditions, are not available [21]. Concurrently, there are many other health-related databases, particularly disease registries that contain disease-specific clinical data [22]. For instance, the Renal Epidemiology and Information Network (REIN) registry records information on all patients with End-Stage Renal Disease (ESRD) who start Renal Replacement Therapy (RRT) in France [23]. The REIN registry includes data on the patient and center’s identification, primary renal disease, initial clinical characteristics, comorbidities, and modalities of ESRD management. Conversely, it does not contain data on healthcare use. However, for researchers, it is important to combine data from the REIN and SNDS databases to assess the patients’ healthcare trajectory, for pharmaco-epidemiological studies, and for health economic analyses. Therefore, the REIN registry routinely produces indicators based on the linkage of its data with those of the SNDS, after approval by the appropriate French authorities.

In line with the GUILD [1] recommendations, here, we describe the fast and efficient record linkage approach used to link patients in the REIN registry with patients in the SNDS database. Then, we illustrate the value of record linkage for epidemiological studies in CKD.

## Method

### Data origin and governance

#### Data from the REIN registry

The REIN registry was launched in 2002 and since 2012, covers the whole French territory. The registry collects data on all patients with ESRD [23] when they start their first RRT (dialysis or preemptive kidney transplantation) for epidemiological purposes. It includes data on the RRT center, the patient’s identification (age, sex, and postcode of the place of residence), comorbidities (e.g., cardiovascular diseases, diabetes, cancer…) and on the first RRT (e.g., date, planned or emergency dialysis…). Patients are followed annually, and specific events are recorded (transplantation, death…) on occurrence. Data collection has been approved by the French National Commission for Information Technology and Privacy (CNIL, N° 903,188), and patients are informed about their inclusion in the REIN registry. Data manually recorded from each dialysis center are then centralized in a national database where a record number, exclusive to the REIN registry, is given to each patient. The Agence de la biomédecine, a public institution, is REIN coordination body. At the end of May 2019, 188,000 patients and 860,000 events were recorded in the REIN database.

#### Data from the SNDS database

The SNDS database is a medicoadministrative database that gathers data from two main sources: i) the reimbursement of ambulatory healthcare procedures (e.g., consultations, biological tests, drug prescriptions…) and ii) hospital activity (i.e., inpatient and outpatient stays). The SNDS database covers around 99% of the French population [21]. Before reaching the central SNDS database, data are pseudonymized and no direct identifier is available. The French legislation allows access to the SNDS database by a restricted number of health-related institutions, including the Agence de la biomédecine. Researchers can have access to data after approval of their research project.

#### Population eligibility

All patients included in the REIN registry were eligible for linkage and constituted the first dataset. For the presentation of our algorithm in this article, all patients with ESRD who started RRT between January 1, 2014 and December 31, 2015 were included. For the SNDS dataset, patients were extracted from the SNDS based on specific treatments (e.g., renal transplantation, immunosuppressive drug prescription, hemodialysis, peritoneal dialysis…) and on hospital stay diagnoses related to CKD, between 2006 and 2016. The date of the first and last known dialysis and the date of renal transplantation (if applicable) were then searched for that period of time. Patients from the SNDS dataset were then categorized according to the year of ESRD incidence (2014 and 2015 for this study).

### Linkage strategy

There are two main record linkage strategies: deterministic and probabilistic. Deterministic strategies are based on a set of matching rules for selected identifiers (i.e., an algorithm). A record pair will only be considered to match if the two records agree on all identifiers of the rules [24].

An iterative and deterministic approach was used to link patients with ESRD in the REIN registry to patients in the SNDS database. The algorithm includes 24 matching rules, or steps, with progressively less strict conditions (Table 1). The algorithm was implemented through a two-phase procedure. First, patients who received a renal transplant (i.e., patients with a renal transplantation date recorded in the REIN registry) were matched to the SNDS database. Then, patients undergoing dialysis and not having received a kidney graft yet and patients with kidney transplant who could not be matched during the first phase were linked. The algorithm and its steps were the same in the two phases, and only the date of treatment (DT) changed. In the first phase, DT was the kidney transplantation date, and in the second phase, DT was the date of the first dialysis for incident patients with ESRD or of the last known dialysis for prevalent patients. The two-phase process is useful because of the fact that the recording of a kidney transplantation date in the two databases (REIN and SNDS) is more reliable and has greater discriminatory power than a date of long-term dialysis.

**Table 1:** Algorithm steps

Step	Required match between the REIN and SNDS datasets	
	Exact	Partial
1	Age, sex, postcode, center ID^a^, death^b^, DT^c^	
2	Age, sex, postcode, center ID, death	DT (− 1 month)
3	Age, sex, postcode, death, DT	center ID (department)
4	Age, sex, postcode, death	center ID (department) DT (−1 month)
5	Age, sex, center ID, death, DT	postcode (department)
6	Age, sex, center ID, death	postcode (department) DT (−1 month)
7	Age, sex, death, DT	center ID (department) postcode (department)
8	Age, sex, death	center ID (department) postcode (department) DT (− 1 month)
9–12	Repetition of steps 2, 4, 6, 8	DT (−2 months)
13	Age, sex, center ID, death, DT	
14	Age, sex, center ID, death	DT (−1 month)
15	Age, sex, death, DT	center ID (department)
16	Age, sex, death, DT	DT (−1 month) center ID (department)
17–24	Repetition of steps 1–8	age (−1 year)

^a^Center ID = Geographical localization number, and then legal entity affiliation number

^b^Death = 3 scenarios: death in both datasets, no death in both datasets, and death recorded only in the REIN dataset

^c^DT = Date of treatment (month/year), renal transplantation during the first phase procedure, dialysis during the second procedure phase

As no unique and direct identifier was available in the two databases, identifiers common to both databases were used in the algorithm: patient’s sex, age, and residence postcode at the time of the considered RRT event, RRT center identification number, month and year of the RRT event (renal transplant, first dialysis, or end-point), and month and year of the patient’s death.

Table 1 describes the algorithm steps and its matching criteria. After a content analysis of the two datasets, particularly how some variables were recorded, less restrictive criteria were added to the algorithm.

Specifically, the SNDS database includes data on hospital stays with a discharge date. These hospital stays are sometimes long and the DT (month) may be different from the discharge date (e.g., dialysis performed at the end of April and hospital discharge in early-mid May). Conversely, the REIN registry records the exact DT. This may lead to a gap in the DT (month) between datasets that must be taken into account (i.e., moving the SNDS DT back of a month; steps 2, 4, 6, 8, 14, and 16).

For the same reason, the patient’s age can be different between datasets. Indeed, in the SNDS database, age is recorded as the age at hospital admission and the birthday date may fall during the hospital stay Therefore, the patient’s age could be n + 1 year in the REIN registry (i.e., subtract a year from the age recorded in REIN; steps 17–24).

During each step, the patient’s death (month and year, then year alone) is taken into account as a matching condition. As in the SNDS database, only death during the hospital stay is available, three matching scenarios were considered: death in both datasets, no death in both datasets, and death recorded only in the REIN registry.

In France, all hospitals and healthcare centers are identified by two numbers. One identifies their geographical localization and the other one their legal entity. As one legal entity number can be associated with several geographical localization numbers, it has a smaller discriminative power. During each step, the RRT center was first considered based on its geographical localization and then based on its legal entity.

Finally, the patient’s place of residence and the treatment center were both considered, first, at their most precise geographical level (i.e., postcode and geographical ID number of the center) and then, at a broader geographical level (department).

The patients’ characteristics between matched and unmatched patients were compared using Chi-square test (Table 3). All record linkages were then evaluated using the patient diabetes status that is recorded in both databases and by calculating agreement statistics (Table 4). The diabetes status of the matched patients in the SNDS database was determined by checking whether drugs used for diabetes treatment were listed in the SNDS database between 2006 and the DT(at least three reimbursements for antidiabetic drug during the year before RRT).. In the REIN registry, the diabetes status was extracted from the nephrologist’s clinical statement (antidiabetic drugs or two blood sugar measurements at the start of RRT ≥ 1,26 g/l fasting or 2 g/l postprandial).

## Results

Among the 22,073 patients in the REIN registry who started RRT between 2014 and 2015, 19,223 (87.1%) were matched, and 2850 (12.9%) did not have any match (Table 2). In the SNDS dataset, 28,402 patients were identified as possible candidates for matching with patients in the REIN registry.

**Table 2:** Number and cumulative percentages of patients matched at each steps, during phase 1 and phase 2 of the linkage process

	Phase 1		Phase 2		Global	
	Patients who received a kidney transplant between 2014 and 2015 (*N* = 2889)		Patients who started dialysis between 2014 and 2015, and phase 1 unmatched patients (*N* = 19,184)		Total patients who started RRT between 2014 and 2015 (*N* = 22,073)	
Steps	N (%)	Cumulative %	N (%)	Cumulative %	N (%)	Cumulative %
1	1216 (42.1)	42.1	8776 (45.7)	45.7	9992 (45.3)	45.3
2	887 (30.7)	72.8	2142 (11.2)	56.9	3029 (13.7)	59.0
3	122 (4.2)	77.0	1283 (6.7)	63.6	1405 (6.4)	65.4
4	85 (2.9)	80.0	263 (1.4)	65.0	348 (1.6)	66.9
5	179 (6.2)	86.2	693 (3.6)	68.6	872 (4)	70.9
6	117 (4)	90.2	179 (0.9)	69.5	296 (1.3)	72.2
7	8 (0.3)	90.5	734 (3.8)	73.3	742 (3.4)	75.6
8	12 (0.4)	90.9	320 (1.7)	75.0	332 (1.5)	77.1
9–12	56 (1.9)	92.8	466 (2.4)	77.4	522 (2.4)	79.5
13	41 (1.4)	94.3	541 (2.8)	80.3	582 (2.6)	82.1
14	24 (0.8)	95.1	171 (0.9)	81.2	195 (0.9)	83.0
15	12 (0.4)	95.5	489 (2.5)	83.7	501 (2.3)	85.2
16	0 (0)	95.5	53 (0.3)	84.0	53 (0.2)	85.5
17–24	4 (0.1)	95.6	350 (1.8)	85.8	354 (1.6)	87.1
Total matched	2763 (95.6)		16,460 (85.8)		19,223 (87.1)	
Unmatched	126 (4.4)		2724 (14.2)		2850 (12.9)	

The first matching phase concerned 2889 patients from the REIN registry with a renal transplantation date, among whom 2763 (95.6%) were matched with a patient in the SNDS dataset. For the second phase, the 126 unmatched patients were added to the 19,058 patients who started dialysis between 2014 and 2015 and without kidney transplantation record (total *n* = 19,184 patients). During this phase, 16,460 (85.8%) patients were matched with one SNDS patient.

The first two steps of the algorithm alone allowed matching 72.8% of patients during the first phase, and 56.9% during the second phase. Conversely, some of the algorithm steps retrieved only about 1% of matches (e.g., steps 14 and 16). Taking into account the one-year difference of patient’s age between datasets (steps 17 to 24) allowed the retrieval of 354 (1.6%) matches.

In total, 310 (1.6%) pairs were not unique matches (e.g., one REIN patients for two SNDS patients) and were chosen at random between competitive matches.

Comparison (univariate analysis) of the characteristics recorded in the REIN registry for the matched (*N* = 19,223) and unmatched patients (*N* = 2850) (Table 3) gave *p*-values below 0.05 due to the population size. However, in terms of raw percentages, the main demographic characteristics (age and sex) were mostly similar between groups, as well as the type of renal disease. On the other hand, the percentage of patients who started RRT by peritoneal dialysis was higher in the unmatched than in the matched group (18.4% versus 9.2%). Similarly, differences were observed concerning the geographical localization of the RRT center. Particularly, the unmatched population included a greater percentage of patients who started RRT in the Auvergne-Rhônes-Alpes region (20.7% versus 10.2% of the matched group). A sub comparison was done including only dialysis patients that did not receive a renal transplant.

**Table 3:** Comparison of the baseline characteristics (extracted from the REIN registry) of matched and unmatched patients with incident ESRD (2014 and 2015)

	Unmatched patients	All matched patients		All matched dialysis patients	
	*N* = 2850	*N* = 19,223		*N* = 15,057	
	n (%)	n (%)	*p*-value^a^	n (%)	*p*-value^a^
Age group					
00–19	28 (1)	227 (1.2)	< 0.001	16 (0.1)	< 0.001
20–44	193 (6.8)	1767 (9.2)		623 (4.1)	
45–64	703 (24.7)	5164 (26.9)		3270 (21.7)	
65–74	690 (24.2)	4671 (24.3)		3934 (26.1)	
75–84	867 (30.4)	5430 (28.2)		5263 (35.0)	
≥ 85	363 (12.7)	1954 (10.2)		1951 (13.0)	
Sex (Women)	1096 (38.5)	6948 (36.1)	0.01	5452 (36.2)	0.07
Diabetes (yes)	1282 (45)	8234 (42.8)	0.02	7372 (49.4)	0.1
Missing	33 (1.2)	167 (0.9)		124 (0.8)	
Cardiovascular diseases			< 0.001		0.028
None	991 (34.8)	7576 (39.4)		5146 (34.2)	
At least 1	1697 (59.5)	10,198 (53.1)		9330 (62.0)	
Missing	162 (5.7)	1449 (7.5)		581 (3.9)	
Primary renal disease			< 0.001		< 0.001
Glomerulonephritis	267 (9.4)	2296 (11.9)		1347 (9.0)	
Vascular nephropathy	788 (27.6)	4988 (25.9)		4464 (29.7)	
Diabetic nephropathy	589 (20.7)	4277 (22.2)		3811 (25.3)	
Other or Unknown	1200 (42.1)	7652 (39.8)		4349 (28.9)	
Missing	6 (0.2)	10 (0.1)		0 (0)	
First RRT			< 0.001		< 0.001
Hemodialysis	2291 (80.4)	16,623 (86.5)		13,833 (91.9)	
Peritoneal dialysis	524 (18.4)	1768 (9.2)		1224 (8.1)	
Renal transplantation	29 (1)	822 (4.3)		0 (0)	
Missing	6 (0.2)	10 (0.1)		0 (0)	
RRT initiation condition			< 0.001		< 0.001
Planned start	1668 (58.5)	12,025 (62.6)		9580 (63.6)	
Emergency start	933 (32.7)	5335 (27.8)		4633 (30.8)	
Missing	249 (8.7)	1863 (9.7)		844 (5.6)	
Treatment center region			< 0.001		< 0.001
Auvergne Rhone Alpes	591 (20.7)	1962 (10.2)		1479 (9.8)	
Bourgone Franche Comté	122 (4.3)	744 (3.9)		596 (4.0)	
Bretagne	200 (7)	715 (3.7)		525 (3.5)	
Centre-Val de Loire	69 (2.4)	775 (4)		587 (3.9)	
Corse	6 (0.2)	81 (0.4)		70 (0.5)	
Grand Est	246 (8.6)	1783 (9.3)		1502 (10.0)	
Hauts de France	174 (6.1)	1907 (9.9)		1640 (10.9)	
Ile-de-France	493 (17.3)	3396 (17.7)		2519 (16.7)	
Normandie	119 (4.2)	961 (5)		732 (4.9)	
Nouvelle Aquitaine	131 (4.6)	1690 (8.8)		1291 (8.6)	
Occitanie	221 (7.8)	1704 (8.9)		1332 (8.9)	
Pays de la Loire	87 (3.1)	894 (4.7)		622 (4.1)	
Provence-Alpes-Côte d’Azur	189 (6.6)	1779 (9.3)		1433 (9.5)	
Overseas territories	196 (6.9)	822 (4.3)		729 (4.9)	

^a^Chi-square test

After linkage completion, the diabetes status of the patients from the REIN registry was compared with that of their SNDS match (Table 4). Among the 19,223 linked patients from the REIN registry, 17,256 (89.8%) shared the same diabetes status in both databases: no diabetes (*n* = 9682; 50.4%) and yes diabetes (*n* = 7574; 39.4%). Cohen’s kappa coefficient was 0.82. Diabetes status discrepancies were observed for 1800 (9.3%) linked patients: 660 (3.4%) had diabetes according to the REIN registry, but not for the SNDS database, and 1140 (5.9%) did not have diabetes for the REIN registry, but did for the SNDS database.

**Table 4:** Diabetes status of patients in the REIN registry and their SNDS matches

Diabetes status of SNDS patients	Diabetes status of REIN patients N (%)			
	No	Yes	Missing	Total
No	9682 (50.4)	660 (3.4)	112 (0.6)	10,454 (54.4)
Yes	1140 (5.9)	7574 (39.4)	55 (0.3)	8769 (45.6)
Total	10,822 (56.3)	8234 (42.8)	167 (0.9)	19,223 (100)

## Discussion

Record linkage is a tool that is increasingly used for public health research worldwide [1–3, 16–19]. In France, to our knowledge, REIN is the first registry to describe its record linkage methodology with the French national health administrative SNDS database.

Our iterative deterministic approach is similar to the one used in other international linkage projects, such as the Clinical Practice Research Datalink (CPRD) in the UK in which primary care data are linked with other patient data [25], and the US Surveillance, Epidemiology and End Results (SEER)-Medicare in which cancer registry data are linked to insurance claims [16]. Differently from these projects, our approach could not use a unique and direct identifier (i.e., social security number or National Health Service number). Despite the lack of this unique identifier, our deterministic approach still gave a good linkage rate (87.1% versus 100% for CPRD [25]).

Compared with probabilistic methods, our deterministic approach is very fast (computation time did not exceed 1 min), despite the use of large datasets (more than 20,000 records in the present example). Zhu and al., demonstrated that deterministic linkage is a better choice in terms of resource efficiency when working with large, good-quality datasets (i.e., less than 5% of missing data and errors for each linkage variable) [26]. In both REIN and SNDS databases, the missing data rate did not exceed 2% for all the variables used for the linkage. Although the error rate is harder to quantify, REIN registry entries are routinely controlled by 36 clinical research assistants throughout its network. Concerning the SNDS data, the French national insurance body and physicians from the medical information department of the different hospitals regularly monitor the validity of the produced data, through samples. Additionally, probabilistic methods are more suitable for linkage based on addresses and names that are prone to specific administrative errors (e.g., misspelling) [27]. These identifiers were not available in our case, strengthening the resort to a deterministic method.

### Flexibility of the iterative deterministic approach

Another advantage inherent to the iterative nature of our approach is the flexibility in the algorithm construction. The key is to consider first the most precise identifiers, and then move progressively towards less stringent matching criteria, thus giving a pyramidal shape to the algorithm. Moreover, the order in which the steps are sequentially put together can be modified, and new steps can be easily introduced.

The need for such flexibility is based on a preliminary analysis of the data to be linked. Indeed, before the implementation of the record linkage, such analysis is highly recommended to ensure high-quality linkage. Specifically, the origin of the linkage variables between databases and their specificities should be rigorously evaluated. In this way, specific data management can be carried out and specific matching rules can be created, enhancing the overall record linkage process. For example, in our case, a patient with ESRD may start dialysis in an intensive renal care unit and then be transferred to another, less medicalized dialysis center for long-term RRT. Therefore, the center recorded in the REIN registry could be the one for long-term RRT and not the intensive unit. However, the intensive unit will be the first dialysis center retrieved from the SNDS dataset, and used for the linkage. Similarly, using the exact DT (day/month/year) might influence the record linkage process. Allowing less stringent matching criteria in the algorithm at a later stage (e.g., using the center department instead of precise geographical ID, or introducing some lag in the DT) helps to retrieve more patients. This preparation time can arguably be considered the most important and critical part of the record linkage process, independently of the used record linkage methodology.

We think that our iterative and sequential approach could be used by many other groups working with health data. Indeed, another advantage of our approach is the low resources, in terms of information, required to obtain a relative high linking rate. The number of linkage variables used in our approach (age, sex, residence, treatment center, treatment date, death date) is relatively small. Moreover, these variables are common data shared by many registries, and can be considered *core variables*. The core of our methodology can be enriched with other linkage variables, in function of the available data and the studied public health issue.

### The value of record linkage for ESRD research

During the last decade, many studies based on the REIN registry have been published, ranging from CKD epidemiology to ESRD outcomes (morbidity and mortality) and spatial analyses. Linking the REIN data with nationwide healthcare data broadens the research perspectives. For instance, the link between prescription of specific drugs and the outcomes following RRT can now be retrospectively assessed. Indeed, our linkage method has been recently used to investigate the effect of angiotensin-converting enzyme inhibitors/angiotensin receptor blockers and beta-blockers in all-cause mortality of incident patients with ESRD without cardiovascular disease [28]. It can also help to compare the validity of registries by comparing the information recorded in two databases [29]. Another ongoing study investigates the link between the pre-RRT care trajectory (consultations with nephrologist and general practitioner), which can be extracted from the SNDS database, and emergency start dialysis, which is recorded in the REIN registry.

### Record linkage evaluation

The main limitation of this approach (and of record linkage in general) is the absence of a gold standard to evaluate the quality of the linkage results. Indeed, two error types can occur with record linkage: true non-matches classified as matches (false positives) and true matches classified as non-matches (false negatives). Typical quality measures include sensitivity, specificity, positive and negative predictive value (PPV, NPV) and f-measure (harmonic mean of sensitivity and PPV) [1, 24, 26].

To measure sensibility, pairs could be manually reviewed to obtain a gold standard. However, such process is extremely time-consuming, because it involves going through the patient’s medical records to compare them with the SNDS data. Moreover, it would require additional legal authorizations and, arguably, a disproportionate logistic deployment. In Australia, Boyd et al., compared their linkage of national morbidity-mortality data with the linkage produced by well-established state-based regional linkage facilities (the gold standard) that use extensive manual reviews [30]. To validate different deterministic linkage rules between a registry and Medicare administrative data, Setoguchi et al. defined their gold standard as the results of a matching rule containing both direct (beneficiary ID) and indirect identifiers (hospital admission date, hospital ID) [31].

Comparing the characteristics of matched and unmatched patients is another way to evaluate the record linkage by identifying a potential selection bias (i.e., a specific population could be left out by the algorithm) [27]. Because our indirect linkage is based on the date of dialysis start and first dialysis facility declared in the REIN registry, some discrepancies between the two sources may be due to the initial period that for some patients may be “blurry” with episodes of acute kidney failure on a chronic decrease of renal function. As well, the dialysis care offer is very dispersed with various providers specialized in different modalities of treatment. For example, dialysis facilities that provide PD at home (private not for profit) are not the same that those who take patients for emergency start (public hospital based). However, our comparison showed that our linkage approach did not lead to any major selection bias. Therefore, researchers using REIN-SNDS linked data can be sure that no specific population will be excluded among patients with ESRD undergoing RRT, regardless of the study objective.

A more accessible validation method (i.e., to determine whether a pair is a true match) is through comorbidities. After record linkage, comorbidities recorded in the registry can be compared with the comorbidity-related healthcare resource usage recorded in the healthcare database. Diabetes appears to be a good indicator. As shown by our results, among the linked patients, around 90% were true matches (i.e., a patient recorded as diabetic in REIN was linked to a patient with diabetes-related treatment in the SNDS). Conversely, around 10% of matches had a more uncertain status. Indeed, these could be false-positive matches. Alternatively, some patients recorded as having type 2 diabetes in the REIN registry might not have needed diabetes medication, and thus were not considered diabetic in the SNDS database.

## Conclusions

The validation of our record linkage methodology is still an on-going process. Describing the record linkage process to inform researchers who will use the resulting data was the first step and the main aim of this article. Researchers using linked REIN-SNDS data can now quote this article to explain the data origin. Transparency, as highlighted in recent guidelines, is the driving force to strengthen the confidence in research using linked data.

## Data Availability

The restrictions due to French Personal data protection regulation (CNIL) prohibit the authors from making the minimal data set publicly available. The access to the data of the REIN registry is governed by a charter. It implies the approval by the REIN scientific board which analyses each request. Information about the data of the REIN registry can be requested by mail to Dr. Cécile Couchoud who manages the REIN registry at the French Biomedicine Agency (cecile.couchoud@biomedecine.fr).
